# A Donor Quality Index for liver transplantation: development, internal and external validation

**DOI:** 10.1038/s41598-018-27960-7

**Published:** 2018-06-29

**Authors:** Audrey Winter, Cyrille Féray, Etienne Audureau, Daniel Azoulay, Corinne Antoine, Jean-Pierre Daurès, Paul Landais

**Affiliations:** 1 Department of Biostatistics, UPRES EA2415, Clinical Research University Institute, Montpellier, France; 2 Beau Soleil Clinic, Montpellier, France; 3 Department of Radiological Sciences, University of California, Los Angeles, CA USA; 4 Department of Hepatology, Henri Mondor Hospital, Créteil, France; 5 Department of Public Health, Henri Mondor Hospital, Créteil, France; 6 Department of Surgery, Henri Mondor Hospital, Créteil, France; 7 Agence de la Biomédecine, Saint-Denis, France

## Abstract

Organ shortage leads to using non-optimal liver grafts. Thus, to determine the graft quality, the Donor Risk Index and the Eurotransplant Donor Risk Index have been proposed. In a previous study we showed that neither could be validated on the French database. Our aim was then dedicated to propose an adaptive Donor Quality Index (DQI) using data from 3961 liver transplantation (LT) performed in France between 2009 and 2013, with an external validation based on 1048 French LT performed in 2014. Using Cox models and three different methods of selection, we developed a new score and defined groups at risk. Model performance was assessed by means of three measures of discrimination corrected by the optimism using a bootstrap procedure. An external validation was also performed in order to evaluate its calibration and discrimination. Five donor covariates were retained: age, cause of death, intensive care unit stay, lowest MDRD creatinine clearance, and liver type. Three groups at risk could be discriminated. The performances of the model were satisfactory after internal validation. Calibration and discrimination were preserved in the external validation dataset. The DQI exhibited good properties and is potentially adaptive as an aid for better guiding decision making for LT.

## Introduction

Liver transplantation (LT) is the major therapy to cure end stage liver disease and hepatocellular carcinoma. In France, the “Agence de la Biomédecine” (ABM) is responsible for managing the waiting list and distributing the grafts. A major impediment to LT is the persistent shortage of donors. Although donor age is one of the strongest risk factor of graft failure^1–3^, the use of grafts from elderly donors has become common. In France, the number of donors older than 65 rose by a factor of 20 between 1998 and 2014. This is partly due to the increase of vascular causes of death and the decrease of traumatic death since 2005.

To assess the quality of a graft, scores such as the Donor Risk Index (DRI) or the Eurotransplant Donor Risk Index (ET-DRI)^1,2^ have been proposed. These two scores appeared to have an impact on the post transplantation survival^1,2^. However, they could not be validated on the French LT database^4–6^. Major donor and recipient differences observed between those databases may explain this result^4,5^. If re-calibrating a model can be considered^7,8^, however, the discrimination cannot be altered^6^. Thus, the current DRIs could not be used on our dataset.

A general policy is to try to give the best organs to the more severe patients with liver failure^3^. Looking for the best match between a graft and its recipient requires preliminary information about the graft quality prior to LT. A donor quality index (DQI), which scores the characteristics of the graft prior to LT, is one of the key factors for a better matching to a recipient. The covariates used to compose the DQI are those available at the time of the harvest in order to define the intrinsic characteristics of the graft. More, in order to better orient the decision of using a graft, a liver donor quality index is of importance. In 2014, in the United-States, 9.6%^9^ of donor livers were not transplanted. This rate was 6.7% in France for our study period (2009–2013) (Supplementary Tables S1 and S2 provide reasons why some grafts were not collected and why some collected organs were not transplanted during the study period, respectively). Moreover, the DQI may be of interest for choosing which graft might benefit from novel preservation techniques.

Since we showed that the existing DRIs did not fit to the French database^4,5^, the primary objective of the present study was to generate a DQI and to perform an internal and external validation.

## Material

### Derivation Dataset

Information relating to LT performed in France between January 4, 2009 and December 31, 2013 was obtained from the ABM (https://www.agence-biomedecine.fr/Organes). The study was conducted with the approval of the Independent Ethics Committee (L 1121-1 to L 1126-11 articles of the Public Health Code). Authorization was also obtained from the “Commission Nationale de l’Informatique et des Libertés” (agreement No. 915206). The data provided were de-identified beforehand.

**Figure 1:**
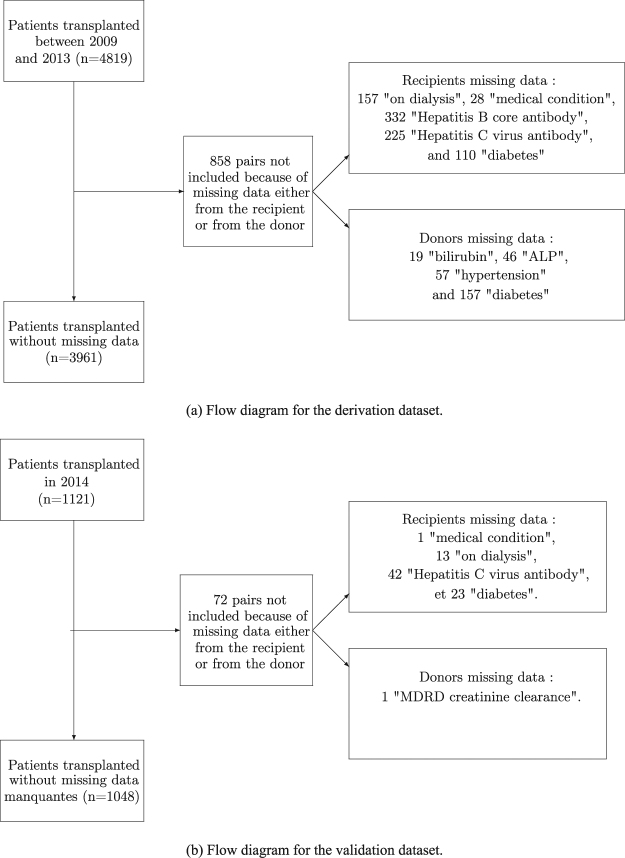
Flow diagrams detailing missing data for recipient transplanted between January 2009 and December 2013 and their donor, in the derivation database and for recipient transplanted in 2014 and their donor, in the validation database.

In accordance with previous works^1,2^, recipients under 18 years, and recipients of multiple organ transplants were not included. Of note, no donation after cardiac death was performed during the retained period (https://www.agence-biomedecine.fr/annexes/bilan2016/donnees/organes/01-prelevement/synthese.htm#figP1). The recipients’ follow-up began at LT and ended with the occurrence of one of the following events: lost to follow-up, death, graft-loss (re-transplantation) or at the end of the study, as of December 31, 2014. The outcome was death or graft-loss. Patients with incomplete covariates were not retained as specified in the flow diagram presented in Fig. 1. Finally, 3961 LT were analyzed. Donor and recipient characteristics are shown in Table 1. This Table contains all the covariates included in the DRI and ET-DRI, and available in the French database^1,2,4^.

**Table 1:** Donor and recipient characteristics with Log-rank test p-value for qualitative variables and quantitative variables across risk groups when significant threshold was met.

Donor characteristics		Mean survival (sd)	P-value
Sex (%):			0.37
Male	55.01	4.55 (0.05)	
Female	44.99	4.56 (0.08)	
Cause of death (%):			**<0.01**
Cerebrovascular accident	60.29	4.44 (0.05)	
Trauma	24.64	4.69 (0.07)	
Anoxia	12.02	4.80 (0.11)	
Other	3.05	4.72 (0.21)	
Diabetes (%):			0.98
yes	7.75	4.56 (0.14)	
no	92.25	4.56 (0.04)	
ABO group (%):			0.22
A	44.58	4.57 (0.06)	
B	9.87	4.72 (0.12)	
O	41.93	4.49 (0.06)	
AB	3.61	4.78 (0.19)	
Hypertension (%):			0.21
yes	35.70	4.49 (0.07)	
no	64.30	4.60 (0.05)	
Malignancy (%):			0.43
yes	1.89	4.72 (0.26)	
no	98.11	4.49 (0.04)	
Alcohol (%):			0.53
yes	15.02	4.50 (0.10)	
no	84.98	4.57 (0.04)	
Smoking (%):			0.47
yes	37.59	4.59 (0.06)	
no	62.41	4.53 (0.05)	
Drugs (%):			0.52
yes	4.17	4.65 (0.19)	
no	95.83	4.56 (0.04)	
Hepatitis C virus antibody (%):			0.46
+	0.45	4.09 (0.56)	
−	99.55	4.53 (0.04)	
Hepatitis B core antibody (%):			0.62
+	4.39	4.61 (0.18)	
−	95.61	4.53 (0.04)	
Inotropes (dobutamine, dopamine, noradrenaline, epinephrine) (%):			0.72
yes	39.16	4.57 (0.06)	
no	60.84	4.55 (0.05)	
Liver type (%):			0.44
Partial/split	5.10	4.43 (0.17)	
Total	94.90	4.55 (0.04)	
Age (%):			**<0.001**
≤69	76.47	4.64 (0.04)	
>69	23.53	4.27 (0.09)	
Height (%):			**0.07**
<162	22.62	4.42 (0.08)	
≥162	77.38	4.60 (0.04)	
Weight, mean (sd)	72.88 (15.17)		
BMI, mean (sd)	25.33 (4.62)		
Sodium: latest (mmol/L) (%):			**0.04**
136–146	43.85	4.47(0.06)	
other	56.15	4.63(0.05)	
Sodium: highest (mmol/L), median (range)	149 (120–180)		
MDRD creatinine clearance: latest (ml/min/1.73 m^2^) (%):			**0.01**
<60	25.30	4.38 (50.08)	
60–89	29.51	4.55 (0.07)	
≥90	45.19	4.66 (0.06)	
MDRD creatinine clearance: lowest (ml/min/1.73 m^2^) (%):			**0.01**
<60	35.35	4.41 (0.07)	
60–89	36.83	4.61 (0.06)	
≥90	27.82	4.68 (0.07)	
Aspartate aminotransferase: latest (U/L), median (range)	39 (0–2000)		
Aspartate aminotransferase: highest (U/L), median (range)	51 (9–2000)		
Alanine transaminase: latest (U/L) (%):			**0.04**
7–41	67.33	4.50 (0.05)	
other	32.67	4.67 (0.07)	
Alanine transaminase: highest (U/L), median (range)	33 (1–2000)		
Total bilirubin: latest (µmol/L), median (range)	10 (0–150)		
Total bilirubin: highest (µmol/L), median (range)	12 (1–150)		
Alkaline phosphatases: latest (U/L) (%):			**0.06**
<33	4.44	4.26 (0.19)	
33–96	76.12	4.54 (0.04)	
>96	19.44	4.71 (0.08)	
Alkaline phosphatases: highest (U/L) (%):			**0.09**
<33	1.78	4.22 (0.31)	
33–96	72.22	4.51 (0.05)	
>96	26.00	4.67 (0.07)	
Gamma glutamyl transpeptidase: latest (U/L), median (range)	30 (0–1477)		
Gamma glutamyl transpeptidase: highest (U/L), median (range)	36 (1–1835)		
Intensive care unit stay (in days) (%):			**<0.001**
≤4	79.68	4.48 (0.04)	
>4	29.32	4.83 (0.08)	
Estimated distance between donor and recipient location (minutes) (%):			**<0.01**
<15	22.60	4.77 (0.08)	
≥15	77.40	4.50 (0.04)	
“Hors tour” (%):			0.84
yes	6.08	4.57 (0.16)	
no	93.92	4.55 (0.04)	
Donor risk index, mean (sd)	1.65 (0.40)	—	—
Eurotransplant donor risk index, mean (sd)	1.63 (0.36)	—	—
UK Donor Liver Index, mean (sd)	1.15 (0.26)	—	—
**Recipient characteristics**		**Mean survival (sd)**	**P-value**
Sex (%):			0.91
Male	73.54	4.55 (0.05)	
Female	26.46	4.56 (0.08)	
Cancer (%):			0.29
yes	29.00	4.62 (0.07)	
no	71.00	4.53 (0.05)	
Decompensated cirrhosis (%):			**<0.001**
yes	37.74	4.73 (0.06)	
no	62.26	4.46 (0.05)	
Non-cirrhotic liver disease (%):			0.63
yes	1.41	4.63 (0.30)	
no	98.59	4.48 (0.04)	
Emergency (%):			**<0.001**
yes	6.72	3.78 (0.17)	
no	93.28	4.58 (0.04)	
MELD exception (%):			0.36
yes	18.00	4.45 (0.09)	
no	82.00	4.57 (0.04)	
Previous transplantation (%):			**<0.001**
yes	8.58	3.76 (0.15)	
no	91.42	4.60 (0.04)	
On dialysis (%):			**<0.001**
yes	5.33	3.43 (0.19)	
no	94.67	4.58 (0.04)	
Medical condition before LT (%):			**<0.001**
Intensive care unit	17.70	3.88 (0.11)	
Hospital (no intensive care unit)	13.88	4.60 (0.10)	
Not hospitalized	68.42	4.71 (0.04)	
Hepatitis B core antibody (%):			0.73
+	20.05	4.53 (0.09)	
−	79.95	4.56 (0.04)	
Hepatitis C virus antibody (%):			**<0.001**
+	23.83	4.23 (0.08)	
−	76.17	4.66 (0.04)	
Diabetes (%):			**0.06**
yes	22.67	4.42 (0.08)	
no	77.33	4.60 (0.04)	
Encephalopathy (%):			**<0.001**
grade 1	68.14	4.62 (0.05)	
grade 2	24.84	4.52 (0.08)	
grade 3	7.02	3.95 (0.17)	
ABO group (%):			0.80
A	45.44	4.56 (0.06)	
B	11.16	4.63 (0.11)	
O	39.03	4.55 (0.06)	
AB	4.37	4.43 (0.19)	
Age, mean (sd)	53.24 (10.39)		
Body mass index (%):			**0.20**
<18.5	3.56	4.35 (0.21)	
18.5–25	44.69	4.50 (0.06)	
≥25	51.75	4.62 (0.05)	
Model for end stage liver disease before LT (%):			**<0.001**
<29	72.73	4.65 (0.04)	
≥29	27.27	4.32 (0.08)	
Waiting time (in days) (%):			**<0.001**
<21	24.54	4.34 (0.08)	
≥21	75.46	4.63 (0.04)	
Follow-up (years), mean (sd)	2.33 (1.63)		

### Validation Dataset

For the external validation, we used 1048 LT performed in France between January 1, 2014 and December 31, 2014, followed-up up to December 31, 2015. Patients with incomplete covariates were not retained (Fig. 1).

## Results

### The DQI

The studied variables and log-rank tests are shown in Table 1. As stated in the methods section, a Cox model with adjustment on recipient characteristics was retained to create the DQI. Several variables (Table 2) were common to the three models. The retained donor’s covariates were: age; cause of death (COD); and intensive care unit (ICU) stay. Several recipient covariates were retained for adjustment: re-transplantation; being on dialysis; status at time of LT; presence of hepatitis C virus antibody; diabetes; and decompensated cirrhosis.

**Table 2:** Retained covariates in the different selection way.

	Covariates	Model 1	Model 2	Model 3	Final model
	**Age**	×	×	×	×
	**Cause of death**	×	×	×	×
Donor	**Intensive care unit stay**	×	×	×	×
	ABO group	×			× (adjustment)
	MDRD creatinine clearance: lowest	×			×
	Partial/split liver	×			×
	Alcohol	×			
	Alkanine phosphatases: latest		×		
	Estimated distance			×	
	**Re-transplantation**	×	×	×	×
	**On dialysis**	×	×	×	×
	**Medical condition before LT**	×	×	×	×
Recipient	**Hepatitis C virus antibody**	×	×	×	×
	**Diabetes**	×	×	×	×
	**Decompensated cirrhosis**	×	×	×	×
	MELD exception	×			×
	ABO group	×			×
	C-index	0.626 (0.009)	0.616 (0.009)	0.616 (0.009)	0.625 (0.009)
Perfomances	K statistic	0.617 (0.007)	0.611 (0.008)	0.610 (0.008)	0.616 (0.007)
	${R}_{D}^{2}$	0.518 (0.020)	0.492 (0.021)	0.492 (0.021)	0.516 (0.021)

Covariates in bold are those which appeared in the three selection models. Model 1: A complete model with selection according to the Akaiké criterion; Model 2: (1) log rank tests with a threshold of 20%, (2) multivariate model included the selected covariates with a selection threshold set at 20%; Model 3: (1) log rank tests with a threshold of 20%, (2) two multivariate models for donor and recipient with the selected covariates with a selection threshold set at 20%, (3) multivariate model with all the covariates selected with a selection threshold set at 20%. The retained full model included all the variables present in at least two of the three models and the set of covariates retained in addition.

The retained full model included all the variables present in at least two of the three models and the set of covariates retained in addition (see methods section) (Table 3): the lowest MDRD creatinine clearance; the liver type; the MELD exception; donor and recipient’s blood groups.

**Table 3:** Retained Cox model also adjusted for: previous transplantation; MELD exception, on dialysis, medical condition before liver transplantation, hepatitis C virus antibody, diabetes, decompensated cirrhosis, recipient’s and donor’s ABO group.

Variables	Estimation of β	HR = exp(β)	SD	Confidence interval 95%	P-value
Age:					
≤69	—	1.00			—
>69	0.28	1.32	0.08	1.14–1.53	<0.01
Cause of death:					
Anoxia	—	1.00			—
Other	0.06	1.07	0.22	0.70–1.63	0.76
CVA	0.30	1.35	0.11	1.08–1.69	<0.01
Trauma	0.11	1.12	0.12	0.88–1.43	0.36
Intensive care unit stay:					
>4	—	1.00			—
≤4	0.24	1.27	0.09	1.07–1.50	<0.01
MDRD creatinine clearance: lowest:					
≥90	—	1.00			—
<60	0.22	1.24	0.08	1.05–1.46	<0.01
60–90	0.05	1.05	0.08	0.89–1.24	0.54
Liver type:					
Total	—	1.00			—
Split	0.39	1.48	0.14	1.11–1.96	<0.01

The liver type covariate was retained since it was present in both DRI and ET-DRI. The MELD exception and donor and recipient’s blood groups covariates were also retained, but for adjustment only.

Hence the proposed DQI score was as follows:

$$\begin{array}{rcl}{\rm{DQI}} & = & \exp \,(0.28\,(1\,{\rm{if}}\,{\rm{donor}}\,{\rm{age}} > 69\,{\rm{years}},\,0\,{\rm{otherwise}})\\  &  & +\,0.06\,(1\,{\rm{if}}\,{\rm{COD}}\,{\rm{is}}\,\mbox{''}{\rm{other}}\mbox{''},\,0\,{\rm{otherwise}})\\  &  & +\,0.30\,(1\,{\rm{if}}\,{\rm{COD}}\,{\rm{is}}\,\mbox{''}{\rm{cerebrovascular}}\,{\rm{accident}}\,({\rm{CVA}})\mbox{''},\,0\,{\rm{otherwise}})\\  &  & +\,0.11\,(1\,{\rm{if}}\,{\rm{COD}}\,{\rm{is}}\,\mbox{''}{\rm{trauma}}\mbox{''},\,0\,{\rm{otherwise}})\\  &  & +\,0.24\,(1\,{\rm{if}}\,{\rm{ICU}}\,{\rm{stay}}\,{\rm{is}}\le \,4\,{\rm{days}},\,0\,{\rm{otherwise}})\\  &  & +\,0.22\,(1\,{\rm{if}}\,{\rm{the}}\,{\rm{lowest}}\,{\rm{MDRD}}\,{\rm{creatinine}}\,{\rm{clearance}}\\  &  &  < \,60\,{\rm{ml}}/{\rm{\min }}/1.73\,{{\rm{m}}}^{2},\,0\,{\rm{otherwise}})\\  &  & +\,0.05\,(1\,{\rm{if}}\,{\rm{the}}\,{\rm{lowest}}\,{\rm{MDRD}}\,{\rm{creatinine}}\,{\rm{clearance}}\\  &  & \ge \,60\,{\rm{ml}}/{\rm{\min }}/1.73\,{{\rm{m}}}^{2}\,{\rm{and}}\, < \,90\,{\rm{ml}}/{\rm{\min }}/1.73\,{{\rm{m}}}^{2},\,0\,{\rm{otherwise}})\\  &  & +\,0.39\,(1\,{\rm{if}}\,{\rm{split}}\,{\rm{or}}\,{\rm{partial}}\,{\rm{liver}},\,0\,{\rm{otherwise}})).\end{array}$$

In Table 4, the progression of the score through different combinations is presented. In our dataset, the average score was 1.83 (SD: 0.44) with a median of 1.76 and a range between 1 and 3.12.

**Table 4:** Examples of some combinations of risk factors and their effect on the score.

Risk factors	Reference patient	Example 1	Example 2	Example 3	Example 4
Age	≤69	**70**	40	60	45
Cause of death	anoxia	**trauma**	**CVA**	**CVA**	**other**
Intensive care unit stay	>4	5	6	**3**	**1**
MDRD creatinine clearance: lowest	≥90	**70**	**82**	**30**	**47**
Liver type	total	total	**split**	total	**split**
Score	1	1.53	2.07	2.13	2.47

### Risk groups

**Figure 2:**
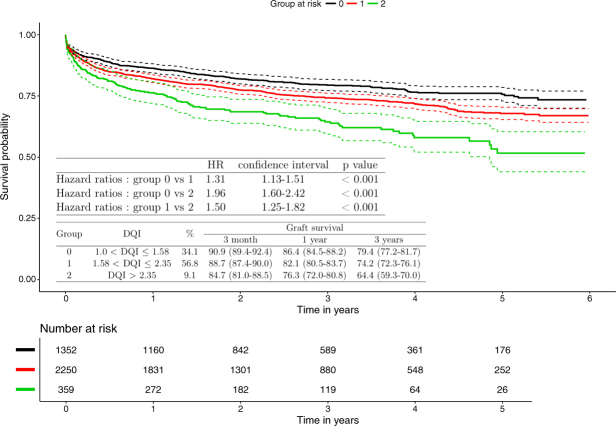
Survival curve using Kaplan Meier estimate for the three risk groups of the DQI score. Graft survival at month 3, year 1 and 3, estimated using Kaplan Meier and hazard ratios through DQI risk groups.

Three groups were obtained according to the following values (Fig. 2): 1.00 < DQI ≤ 1.58; 1.58 < DQI ≤ 2.35 and DQI > 2.35, comprising 34.1%, 56.8% and 9.1% patients, respectively. Three-month, 1-year and 3-year survivals were calculated for each group (Fig. 2). HRs were expressed according to the groups at risk (Fig. 2).

### Calibration plot

The calibration of the score was assessed. As shown in Fig. 1S (see Supplementary Figures) the score seems to slightly underestimate the probability of death/graft loss at all months tested. This underestimation was not significant for all the predicted probabilities since more than 50% of the 95 band of the calibration plot contained the first bisector.

Of note, the 95 band was wide at the higher probability of death/graft loss due to few remaining patients at risk in this category. However, despite a slight underestimation, the longer the follow-up, the lower was the underestimation of the risk of death/graft loss.

### Model performance and internal validation

As the predictive performances of the different models were close (Table 2), the covariates selection was implemented with the simplest method, namely the backward selection using Akaiké criterion. The corrected performances of the model by the bootstrap method were 0.609 (0.591–0.627) for the Harrell C-index, 0.604 (0.590–0.617) for the Gönen and Heller K statistic and 0.464 (0.408–0.520) for the Royston and Sauerbrei ${R}_{D}^{2}$. The confidence intervals were calculated using 200 bootstrap estimates. The performances of the model remained satisfactory even after correction by the optimism.

### External validation

An external validation^4,6^ was performed in the validation dataset, which was constituted of grafted patients in 2014. This dataset was independent of the derivation dataset. To calculate the DQI in the validation dataset, we followed the same procedures as proposed previously in the construction steps. Calibration and discrimination were evaluated through seven steps.

#### Comparing the databases

The comparison of the datasets appears in Table 5. The distribution of the DQI from the derivation and validation datasets is presented in Fig. 2S (see Supplementary Figures). It appears that the DQI is higher in the validation dataset than in the construction dataset. This might be due to older donors and more frequent strokes.

**Table 5:** Differences in donor and recipient characteristics between the derivation and validation datasets (P-values, *χ*^2^ tests and ANOVA when appropriate).

Recipient characteristics	2009–2013 (n = 3961)	2014 (n = 1048)	P- values
Expert component (no)	3248 (82%)	874 (83.4%)	0.31
ABO group			0.59
A	1800 (45.44%)	465 (44.37%)	
AB	173 (4.37%)	38 (3.63%)	
B	442 (11.16%)	118 (11.26%)	
O	1546 (39.03%)	427 (40.74%)	
Re-transplantation (no)	3621 (91.42%)	961 (91.7%)	0.82
On dialysis (no)	3750 (94.67%)	987 (94.18%)	0.58
Medical condition before LT			0.45
Home	2710 (68.42%)	705 (67.27%)	
Hosptial	550 (13.89%)	140 (13.36%)	
ICU	701 (17.7%)	203 (19.37%)	
Hepatitis C virus antibody (−)	3017 (76.17%)	811 (77.39%)	0.43
Diabetes (no)	3063 (77.33%)	793 (75.67%)	0.27
Decompensated cirrhosis (no)	2466 (62.26%)	667 (63.65%)	0.43
**Donor characteristics**	**2009–2013 (n** = **3961)**	**2014 (n** = **1048)**	**P-values**
ABO group			0.75
A	1766 (44.58%)	460 (43.89%)	
AB	143 (3.61%)	32 (3.05%)	
B	391 (9.87%)	102 (9.73%)	
O	1661 (41.93%)	454 (43.32%)	
Age			<0.01
≤69	3029 (76.47%)	710 (67.75%)	
>69	932 (23.53%)	338 (32.25%)	
COD			<0.01
Anoxia	476 (12.02%)	150 (14.31%)	
CVA	2388 (60.29%)	651 (62.12%)	
Trauma	976 (24.64%)	231 (22.04%)	
Other	121 (3.05%)	16 (1.53%)	
ICU stay (in days)			
<4	3156 (79.68%)	853 (81.39%)	
>4	805 (20.32%)	195 (18.61%)	
Clearance MDRD: lowest (ml/min/1.73 m^2^)			
<60	1400 (35.34%)	385 (36.74%)	
60–89	1459 (36.83%)	388 (37.02%)	
>90	1102 (27.82%)	275 (26.24%)	
Liver type (entire)	3759 (94.9%)	1007 (96.09%)	0.13

#### Regression on prognostic index (PI)

The slope on the PI, i.e. ln(DQI), was 0.88 (SD = 0.29). The slope was not different from 1 (P-value = 0.68, likelihood-ratio test); thus, the discrimination was preserved in the validation dataset.

#### Check model misspecification/fit

Since the estimation of the β* coefficients were not different from 0 (p = 0.64), no adjustment on the DQI covariates was needed.

We tested if there was a lack of fit for the adjustment covariates, i.e. recipients’ covariates. The ${\beta }_{adjustment\,}^{\ast }$ were different from 0 (p < 0.01), an adjustment on the adjustment covariates was needed.

The assumption of proportional hazards was verified for all covariates.

#### Measures of discrimination

The Harrell C-index was 0.587 (0.545–0.628), the Gönen and Heller K statistic was 0.612 (0.594–0.630) and the Royston and Sauerbrei ${R}_{D}^{2}$ was 0.416 (0.309–0.523). Discrimination measures on the validation and derivation datasets were close.

#### Kaplan-Meier curves for groups at risk

**Figure 3:**
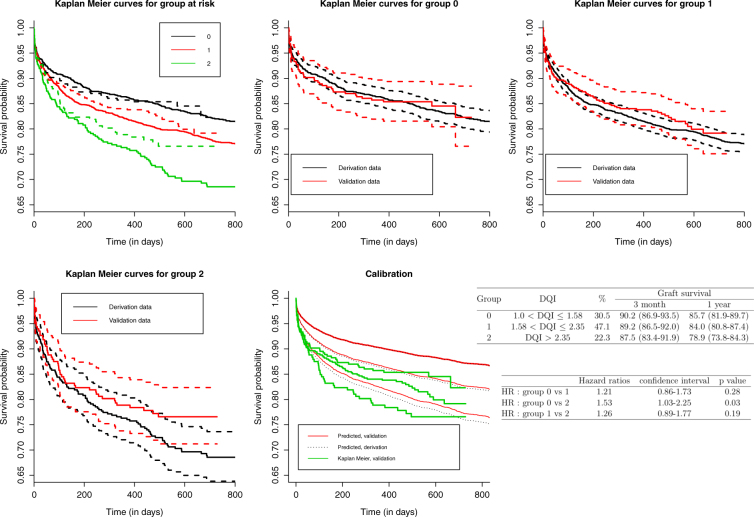
Survival curve using Kaplan Meier estimate for the three risk groups of the DQI score in the derivation (solid lines) and validation (doted lines) datasets. Predicted survival curves in the derivation and validation dataset using the PI and the baseline survival in the derivation dataset with the estimated survival using Kaplan Meier curves in the validation dataset. Graft survival at month 3, year 1 and 3, estimated using Kaplan Meier and hazard ratios through DQI risk groups for validation data.

Failure free survival per DQI categories for derivation and validation datasets is given in Fig. 3. The follow-up in the derivation data was truncated to 800 days.

First, Kaplan Meier curves were well separated. Then, the risk groups were discriminative. Second, Kaplan Meier curves from derivation and validation data were close. For greater accuracy, we then plotted three survival curves for each risk group with confidence intervals in derivation and validation datasets (Fig. 3). Validation curves are within the confidence interval of the derivation dataset curves (except for group 1: from 300 to 500 days and the tail of group 2). The apparent calibration thus seems to be preserved.

#### Hazard ratios between groups at risk

HRs between groups at risk are presented in Fig. 3. They were not significantly different.

#### Calibration

The Kaplan Meier curves (green, Fig. 3, see “calibration”) seem to underestimate survival. The predicted survival curves in the two datasets (red and black) are superimposed for the first risk group, very close for the second risk group and more separated for the third group. Then, there is some similarity in the PI distribution in each risk group in the derivation and validation datasets. The empirical cumulative distribution functions of the PI by dataset and risk group, on Fig. 3S (see Supplementary Figures), further support this.

## Discussion

A DQI, which qualifies the liver graft, is one of the key factors for a better matching between donor and recipient. As the existing DRIs were not discriminant (i.e. slope on the PI: 0.57 (SE 0.15) and 0.64 (SE 0.16) for the DRI and ET-DRI respectively, P-value < 0.001) and miscalibrated (e.g. survival, according to DRIs categories, was not consistent between the construction and validation datasets) according to our dataset^4,5^, because of population differences. We thus decided to create a DQI, which characterizes the graft in view of transplantation. It is based on Cox model adjusted on the recipients’ characteristics. Three different ways to elaborate the model were tested. We explored new covariates that were not taken into account in the DRI and ET-DRI, defining the donor such as MDRD clearance or adjustment covariates such as MELD exception, decompensated cirrhosis or HCC.

Five donor covariates were retained in the final model: age, cause of death, intensive care unit stay, lowest MDRD creatinine clearance, and liver type (split or total).

We obtained three groups of different sizes (Fig. 2). Of note, there is no consensus in the literature regarding selecting the number of risk groups or in positioning the cut-off points to delineate these groups^10^. Too many groups could be unstable and consequently discrimination becomes insufficient. The recommendation is to create three to five groups, not necessarily of the same size, in order to highlight extreme groups^10^. After construction of the risk groups, HRs (Fig. 2), between groups, showed significant differences in mortality/graft loss. Indeed, the curves were all well separated, and the survival decreased with the increase of the score (Fig. 2). The third group will correspond to donors at higher risk that we may define as “extended criteria donors”. As shown by Collett *et al*.^11^, the average MELD scores for recipient in the three groups at risk (20.6, 20.8 and 19.3, respectively) were not different (p = 0.06). The performance of the model remained satisfactory even after correction by the optimism. Finally, an external validation^4,6^ was performed; discrimination and apparent calibration were preserved in the validation dataset.

Our study has some limitations. First, it is very difficult to evaluate the intrinsic quality of a graft since the allocation procedure associates to each donor a recipient. The quality of the graft is a function of the graft/recipient survival. We then assume that the allocation was appropriate. The graft/recipient survival provides an indirect measure of the donor quality and thus can be considered as a surrogate variable. We assumed that this variable was highly correlated to the target variable, i.e. the graft quality, and was thus able to be extrapolated as an outcome.

In prognostic models, discrimination measures are generally not high^12^ which the case in our study. This occurred even when models were built in large datasets^2,13^.

A selection bias has been explored as 858 recipient/donor pairs were removed from the model due to missing data. We compared the distributions of data of this subset to the data used in the analysis either for covariates belonging to the recipient (age, sex, cancer, decompensated cirrhosis, waiting time, medical condition before LT) or donor (age, sex, COD, liver type). After Bonferroni correction, only the presence of a cancer and medical condition before LT were significant (*χ*^2^ tests). Of note, only the covariate “presence of a liver cancer” was not part of the score. However, the frequency of liver cancer was higher in the analysis data set, which limits the occurrence of a potential bias.

As the size was part of the DRI and the weight of donors is often difficult to establish due to fluctuations in hydration during the ICU stay, we gave more importance to the size and we did not include the weight in our study. Nevertheless, we tested the weight in the final model. The weight did not provide a pertinent information to be added in the model (p = 0.82). This result is consistent with the DRI and ET-DRI models^1,2^.

Macro/micro-steatosis was not included in the model since this information was not available in the French database.

Since the cold ischemia time (CIT) is only known at the time of LT, this covariate was not retained as an intrinsic characteristic of the graft in the model, as outlined in^11,14^, even though this covariate has an impact on post LT survival, such as post- and peri-operative period covariates. These covariates are not known at the time of graft procurement and can’t contribute to the creation of a score, which qualifies the donor. The aim of this work was to characterize the donor independently of the potential recipient. Of note, the distance between the organ procurement center and the transplant center was not correlated to the cold ischemia time (Pearson correlation coefficient r = 0.08), indicating that cold ischemia time is highly dependent on the logistic of transplantation. Moreover, the introduction of perfusion machines may completely reverse the paradigm of organ preservation, and thus will modify the impact of the cold ischemia time. In effect, increasing the duration of organ conservation in standardized conditions may consistently improve the chances for a patient to benefit from the most compatible graft. Whatever the case may be, cold ischemia time did not go through the three different ways of selecting the covariates. About the exploration of a potential bias, as CIT is not known at the time of graft procurement, it does not lead to a selection or misclassification bias. However, there may be confounding bias because CIT exerts an effect on the recipient/graft survival. We went back and found that this was not the case. In effect, CIT had no specific weight in the Cox model (HR: 0.9998 [0.9996–1.0001], p = 0.26), therefore we did not include it in the final model even as an adjustment covariate. Adding CIT in the final model as adjustment covariate would have decreased both the quality and discrimination performance of the model (i.e. increase in Akaiké criterion (AIC) and decrease in c-index, respectively). For example, in case-control studies, when a model is forced to include non-retrained covariates, the model often becomes overfitted. An overfitted model has a weak validity and the model would have insufficient discriminative power in new patients.

Donor age was different between the Organ Procurement and Transplantation Network (OPTN) database^1^ and the French database^4,5^. Since age below 70 did not exert a significant effect on recipient survival in our database, the first three quartiles were grouped together. Age over 70 represented 25% in the French database compared to 4.3% in the OPTN database.

For COD, 60% of patients presented a CVA, 12% an anoxia and 25% a trauma. This distribution was quite different from that observed in the OPTN database^1^, which was 44%, 9% and 45%, respectively (p < 0.001). The risk of death/graft loss was increased in patients who experienced a CVA compared to anoxia (HR: 1.35 [1.08–1.69], p < 0.01). This result was consistent with Feng *et al*.^1^ and Singhal *et al*.^15^.

In three out of four stays in ICU the donor’s length of stay was less than 4 days. Shorts stays in ICU were associated with an increased risk of death/graft loss. As outlined above, CVA was associated with an increased risk of death/graft loss compared either to anoxia or trauma COD. Given that the length of ICU stays of donors with a CVA was ≤4 days in more than 80% of cases (which represents 49% of the donors), it supports the fact that short stay in ICU in our series was related to a shorter recipient survival. In the literature the donor ICU stay did not appear to influence graft survival^16,17^. However, Cuende *et al*.^18^ included 3429 LT and showed that an ICU stay of more than six days represented a moderate risk (RR: 1.21 [1.1–1.4]).

Finally, concerning the external validation, the survival among the different DQI groups was not significantly different (HRs with large confidence interval). This may be due to a lack of power given that the follow up was shorter than in the derivation dataset. We were thus truncated the derivation data follow-up. A longer follow-up period would have been beneficial to obtain a better accuracy. Nevertheless, Kaplan Meier curves were well separated (Fig. 3). Then, the risk groups were discriminative. For the calibration step, an under-estimation was observed. This may be due to the specific structure of the DQI. Indeed, this score includes only a part of the PI of the original model (the *β*_*adjustment*_ for recipient covariates were not included). But, baseline survival was calculated on the original model, which includes all the covariates; both donor and recipient. The weights of the recipients’ covariates then may play an important role in the estimation of ${S}_{0}^{deriv}(t)$, i.e. the baseline survival in the derivation dataset. We checked whether this hypothesis was consistent by computing the baseline survival on the derivation dataset, without the recipients’ covariates (Fig. 4S, Supplementary Figures). Compared to those in Fig. 3, the predicted survival was under-estimated. It confirms that baseline survival is affected by recipients’ covariates. This result therefore impacts the seventh step of the external validation. Nevertheless, according to Kaplan Meier curves (Fig. 3), the apparent calibration and the discrimination were preserved in the validation dataset.

In a recent review, Flores and Asrani^14^ suggested that the donor score might benefit from being updated. We aimed to create a flexible DQI model and adaptable. Indeed, if calibration is lacking with a validation dataset comprising a longer follow-up, and if the discrimination is preserved, a re-calibration of the DQI will be possible. This type of procedure is dedicated to be repeated yearly using LT data from subsequent years. This adaptive procedure will enable to gradually take into account the modifications of the graft allocation system. This approach may be of interest for other countries. Indeed, French allocation system is quite similar to most European systems. Moreover, in France, the MELD and MELD exceptions are also used such as in the United States of America. For an international-reader, our model is a tool for decision support in countries with a high activity of harvest of brain dead organ donors, with a significant increase of elderly donors, such as in Italy, Spain or Portugal thanks to a political proactive organ census and collection of old or very old donors. We encourage these countries to make an external validation of the DQI on their own datasets. Furthermore, recently, another national index was introduced, the DLI (Donor Liver Index, which has been calculated in the French dataset (Table 1))^11^. This score is an example that a national index gives complementary information to bring an aid to improving the evaluation of the quality of grafts.

This DQI allows considering a next step to explore the optimal matching between donor and recipient and the “extended criteria donor” graft attribution, to improve the success of LTs, through sequential stratification method^3^. The DQI will be used to qualify the graft. We also aim to create a score based on the survival benefit, which combines two models: a pre-transplantation model and a post-transplantation model. The post-transplantation model takes into account the DQI and integrate the results of the sequential stratification study. A validation will be performed using a discrete-event simulation model.

More, use of machine perfusion protocols are starting this year in France. Until now, the only source of grafts are brain dead donors. These perfusion machines will likely allow rehabilitation of critical grafts and also will offer viability tests, which may eventually be compared to the DQI to test its relevance. Machine perfusion are quite expensive, where such a prognostic score may be of interest to target grafts and who would benefit the most from the infusion.

## Methods

In order to simplify the interpretation of the model, and moreover to protect against outliers, we transformed the quantitative variables into qualitative variables. When there was no clinical threshold generally accepted (contrary to biological covariates such as alkaline phosphatases or Modification of the Diet in Renal Disease (MDRD) creatinine clearance for which thresholds are recommended) we defined four groups according to the quartiles of the covariate distributions. Graft survival curves were plotted according to the product limit method. They were then compared using hazard ratios (HR). In the absence of significant difference between groups, they were re-grouped together. If no groups at risk were identifiable for a given covariate, then this covariate was kept in the model for adjustment as a quantitative variable (or its natural logarithmic form, when appropriate), according to the three models presented below.

### Definitions of covariates

In France, all donors are cared for in intensive care units (ICU). The ICU length of stay is based on the number of nights spent, rather than the number of days. Therefore, a stay of 0 day is observable.

For recipient, decompensated cirrhosis was identified in the database using the MELD score and the Child-Pugh score. All recipients presenting with cirrhosis of the liver, a MELD score ≥16 and a Child-Pugh B or C were considered as decompensated cirrhosis.

Patients first-transplanted, without cancer and cirrhosis, were considered as having a non-cirrhotic liver disease.

The estimated distance between donor and recipient centers was calculated on the basis of a geographic model taking into account road distances in minutes.

MELD exceptions were identified and resulted in extra points while on the waiting list^19^.

The variable named “Hors tour” means that after at least five consecutive refusals by the transplant teams, the graft is then supplied to a transplant team who have identified an appropriate candidate.

### Score creation

In the derivation dataset, as in Feng’s^1^ and Braat’s^2^ studies we used a Cox model with adjustment on recipient characteristics to create the score (Table 1). We tested three different ways to elaborate this model:

1. A complete model with a covariate selection according to AIC;
2. An analysis using a two-step procedure. First, performing log rank tests with a threshold of 20% for the type I error for each covariate, according to the outcome. Then, followed by a multivariate model that included the selected covariates on the basis of the previous step with a selection threshold set at 20% for the type I error.
3. An analysis using the same first step of the procedure above was performed but followed by two multivariate models for donor and recipient, respectively. These models included the selected covariates on the basis of the first step with a selection threshold set at 20% for the type I error. Finally, a multivariate model was set up with all the covariates selected in the previous models with a selection threshold set at 20% for the type I error.

We then retained a model including all the covariates present in at least two of the three models. Moreover, in this model we also tested each covariate, which appeared in only one of the three models. Covariates were retained if their P-values were lower than 5%. Then, the final model included the set of covariates present in two of the three models and the set of covariates retained in addition. This approach prevented omission any relevant covariates.

### Risk groups

To create risk groups, we used 10 groups according to the deciles of the score. We drew survival curves according to the corresponding Kaplan Meier estimates, which were compared according to the HR. In the absence of significant difference between groups, they were thus grouped together.

### Calibration plot

A calibration plot reports graphically predicted outcome probabilities (on the x-axis) against observed outcome frequencies (on the y-axis)^20^. A well-calibrated prediction implies that the curve lies on the first bisector. Thus, for each point, the predicted probability is equal to the observed outcome frequencies. In a Cox model, calibration may not be easily assessed^20^. The model allows estimation of relative risk differences between patients presenting with different characteristics. However, since it does not estimate the baseline survival function, it does not estimate absolute risks (event probabilities), in contrast to parametric models^6^. However, absolute risks can be calculated by focusing on a fixed time point (e.g., risk at month 3). Thus, we plotted three calibration plots at months 3, 6 and 12. We calculated the corresponding baseline survival rates, estimated according to Breslow’s estimator on the complete model^21^. Then we calculated the predicted probabilities of death/graft loss^20^ as:

1 $$1-{S}_{0}{(t)}^{\exp ({\beta }_{1}{X}_{1}+\ldots +{\beta }_{n}{X}_{n})}$$

where *S*_0_(t) is the baseline survival, *β*_*i*_ the donor coefficients of the Cox model and *X*_*i*_ the donor covariates. Note that the estimated parameters of the score did not change according to the baseline survival. Coefficients are those estimated at the previous step.

### Model performance and internal validation

To evaluate the model performance, we calculated several indices: the C-index of Harrell^22^, the K statistic of Gönen and Heller^23^ and the ${R}_{D}^{2}$ of Royston and Sauerbrei^24^. Harrell’s C-index is defined as the proportion of all usable patient pairs for which the predictions and outcomes are concordant^22^. Gönen and Heller’s K statistic is used to evaluate the discriminant power and the predictive accuracy of nonlinear statistical models. It is a function of the regression parameters and the covariates distribution only, and is therefore asymptotically unbiased^23^. Royston and Sauerbrei’s ${R}_{D}^{2}$ is a measure of the proportion of explained variation, based on D, a measure of the ability of a model to discriminate between good and poor patient outcomes^24^.

Internal validation is a necessary part of model development^25^, although according to Moons^20^, internal validation quantifies the predictive ability of a model on the derivation data (often called apparent performance^26–28^). Internal validation was assessed using a bootstrapping procedure in order to quantify the optimism associated with the performance of the model. A seven-step procedure was performed according to the method described in Moons^20^. The confidence intervals of the 3 indices were calculated using 200 bootstrap estimates.

### External validation

We performed an external validation of the DQI according to Royston and Altman^6,24^; the method described in a previous study^4^. The external validation of a model explores the assessment of its performance on an independent database^6^. Model performance was evaluated considering two fundamental aspects: discrimination and calibration. Discrimination, known as “separation”, allows differentiation of patients’ prognoses through risk estimates from the model. Calibration reflects the prediction accuracy; if well-calibrated, a score assigns the appropriate probability at each level of the predicted risk^6^.

#### Comparison of the datasets

A comparison of the construction dataset with the validation dataset was performed using *χ*^2^ tests or ANOVA, when appropriate.

#### Regression on the prognostic index (PI) in the validation data

In order to obtain the prognostic index (PI), we used ln(DQI). The model fitted was as follows:

2 $${\beta }_{DRI}\,\mathrm{ln}\,({\rm{DQI}})+{\beta }_{adjustment}\,{X}_{adjustment}$$

with *X*_*adjustment*_ the covariates used in the DQI and *β*_*adjustment*_ fixed at the estimated value in the original model. The discrimination is considered good when this coefficient is equal to 1 and poor if the slope is lower than 1; a coefficient >1 is considered very good.

In order to test $\hat{{\rm{\beta }}}\,{}_{{\rm{DQI}}}$ = 1, we used a likelihood ratio test.

#### Check model misspecification/fit

A possible reason for a PI coefficient less than 1 is poor adjustment of one or more covariates. To test whether one or more of the DQI covariates needed an adjustment we fitted:

3 $${\beta }_{DQI}\,\mathrm{ln}\,(DQI)+{\beta }_{adjustment}\,{X}_{adjustment}+{\beta }^{\ast }{\boldsymbol{Z}}$$

where ***Z*** were the covariates used to build the DQI.

In this model, the *β*_*DQI*_ was set at 1, and the *β*_*adjustment*_ was fixed at the estimated value in the original model. Next, we performed a likelihood ratio test to test the following equation: β* = 0. The proportional hazards risk assumption was checked using the Schoenfeld residuals.

#### Measures of discrimination

As in the construction step we calculated three discrimination indexes: Harrell C-index^22^, Gönen and Heller K statistic^23^, and Royston and Sauerbrei ${R}_{D}^{2}$ ^24^.

#### Kaplan-Meier curves for groups at risk

We plotted the survival curves according to the groups at risk using the Kaplan Meier estimates. We also did a visual comparison of these curves with those of the construction dataset to evaluate the calibration. Indeed, if the survival curves of the construction and validation data for each group at risk were superimposable, then the visual calibration was considered as preserved. Discrimination can also be evaluated from the curves; the more the survival curves are separate the better the discrimination.

#### Hazard ratios (HR) between groups at risk

HR were estimated for each group at risk using a Cox model. The higher the discrimination, the larger the HR.

#### Calibration

In this step, we estimated the baseline survival in the derivation: ${S}_{0}^{deriv}(t)$ data, using Breslow’s estimator^21^. We then calculated the estimated survival function in the validation dataset as: ${S}^{val}(t,\,DQ{I}_{i})=\,{S}_{0}^{deriv\,}{(t)}^{DQ{I}_{i}}$. For each group at risk, we averaged ${S}_{i}^{val}(t)$, at each time-point, to obtain the expected survival curves. The same procedure was performed for ${S}^{deriv}(t,\,DQ{I}_{i})$. We then superimposed the predicted survival curves for each risk group in the derivation and validation dataset, and the Kaplan Meier curves observed for each group in the validation dataset.

All analyses were performed using R software, version 3.3.0 (R Development Core Team, A Language and Environment for Statistical Computing, Vienna, Austria, 2016. https://www.R-project.org/).

### Data availability

The data that support the findings of this study are available from the French Agency for Biomedecine but restrictions apply to the availability of these data, which were used under license for the current study, and so are not publicly available. According to the French regulation, upon reasonable request, data are however available with permission from the Agency for Biomedicine.

## Electronic supplementary material

Supplementary tables and figures
