# Author Correction: A Donor Quality Index for liver transplantation: development, internal and external validation

**DOI:** 10.1038/s41598-018-30974-w

**Published:** 2018-10-05

**Authors:** Audrey Winter, Cyrille Féray, Etienne Audureau, Daniel Azoulay, Corinne Antoine, Jean-Pierre Daurès, Paul Landais

**Affiliations:** 1 Department of Biostatistics, UPRES EA2415, Clinical Research University Institute, Montpellier, France; 2 Beau Soleil Clinic, Montpellier, France; 3 Department of Radiological Sciences, University of California, Los Angeles, CA USA; 4 Department of Hepatology, Henri Mondor Hospital, Créteil, France; 5 Department of Public Health, Henri Mondor Hospital, Créteil, France; 6 Department of Surgery, Henri Mondor Hospital, Créteil, France; 7 Agence de la Biomédecine, Saint-Denis, France

Correction to: *Scientific Reports* 10.1038/s41598-018-27960-7, published online 29 June 2018

This Article contains errors.

In Table 1, the formatting for the header “Recipient characteristics” is incorrect. The correct Table 1 appears below:

**Table 1:** Donor and recipient characteristics with Log-rank test p-value for qualitative variables and quantitative variables across risk groups when significant threshold was met.

Donor characteristics		Mean survival (sd)	P-value
Sex (%):			0.37
Male	55.01	4.55 (0.05)	
Female	44.99	4.56 (0.08)	
Cause of death (%):			**<0.01**
Cerebrovascular accident	60.29	4.44 (0.05)	
Trauma	24.64	4.69 (0.07)	
Anoxia	12.02	4.80 (0.11)	
Other	3.05	4.72 (0.21)	
Diabetes (%):			0.98
yes	7.75	4.56 (0.14)	
no	92.25	4.56 (0.04)	
ABO group (%):			0.22
A	44.58	4.57 (0.06)	
B	9.87	4.72 (0.12)	
O	41.93	4.49 (0.06)	
AB	3.61	4.78 (0.19)	
Hypertension (%):			0.21
yes	35.70	4.49 (0.07)	
no	64.30	4.60 (0.05)	
Malignancy (%):			0.43
yes	1.89	4.72 (0.26)	
no	98.11	4.49 (0.04)	
Alcohol (%):			0.53
yes	15.02	4.50 (0.10)	
no	84.98	4.57 (0.04)	
Smoking (%):			0.47
yes	37.59	4.59 (0.06)	
no	62.41	4.53 (0.05)	
Drugs (%):			0.52
yes	4.17	4.65 (0.19)	
no	95.83	4.56 (0.04)	
Hepatitis C virus antibody (%):			0.46
+	0.45	4.09 (0.56)	
−	99.55	4.53 (0.04)	
Hepatitis B core antibody (%):			0.62
+	4.39	4.61 (0.18)	
−	95.61	4.53 (0.04)	
Inotropes (dobutamine, dopamine, noradrenaline, epinephrine) (%):			0.72
yes	39.16	4.57 (0.06)	
no	60.84	4.55 (0.05)	
Liver type (%):			0.44
Partial/split	5.10	4.43 (0.17)	
Total	94.90	4.55 (0.04)	
Age (%):			**<0.001**
≤69	76.47	4.64 (0.04)	
>69	23.53	4.27 (0.09)	
Height (%):			**0.07**
<162	22.62	4.42 (0.08)	
≥162	77.38	4.60 (0.04)	
Weight, mean (sd)	72.88 (15.17)		
BMI, mean (sd)	25.33 (4.62)		
Sodium: latest (mmol/L) (%):			**0.04**
136–146	43.85	4.47(0.06)	
other	56.15	4.63(0.05)	
Sodium: highest (mmol/L), median (range)	149 (120-180)		
MDRD creatinine clearance: latest (ml/min/1.73 m^*2*^) (%):			**0.01**
<60	25.30	4.38 50.08)	
60–89	29.51	4.55 (0.07)	
≥90	45.19	4.66 (0.06)	
MDRD creatinine clearance: lowest (ml/min/1.73 m^*2*^) (%):			**0.01**
<60	35.35	4.41 (0.07)	
60–89	36.83	4.61 (0.06)	
≥90	27.82	4.68 (0.07)	
Aspartate aminotransferase: latest (U/L), median (range)	39 (0–2000)		
Aspartate aminotransferase: highest (U/L), median (range)	51 (9–2000)		
Alanine transaminase: latest (U/L) (%):			**0.04**
7–41	67.33	4.50 (0.05)	
other	32.67	4.67 (0.07)	
Alanine transaminase: highest (U/L), median (range)	33 (1–2000)		
Total bilirubin: latest (µmol/L), median (range)	10 (0–150)		
Total bilirubin: highest (µmol/L), median (range)	12 (1–150)		
Alkaline phosphatases: latest (U/L) (%):			**0.06**
<33	4.44	4.26 (0.19)	
33–96	76.12	4.54 (0.04)	
>96	19.44	4.71 (0.08)	
Alkaline phosphatases: highest (U/L) (%):			**0.09**
<33	1.78	4.22 (0.31)	
33–96	72.22	4.51 (0.05)	
>96	26.00	4.67 (0.07)	
Gamma glutamyl transpeptidase: latest (U/L), median (range)	30 (0–1477)		
Gamma glutamyl transpeptidase: highest (U/L), median (range)	36 (1–1835)		
Intensive care unit stay (in days) (%):			**<0.001**
≤4	79.68	4.48 (0.04)	
>4	29.32	4.83 (0.08)	
Estimated distance between donor and recipient location (minutes) (%):			**<0.01**
<15	22.60	4.77 (0.08)	
≥15	77.40	4.50 (0.04)	
“Hors tour” (%):			0.84
yes	6.08	4.57 (0.16)	
no	93.92	4.55 (0.04)	
Donor risk index, mean (sd)	1.65 (0.40)	—	—
Eurotransplant donor risk index, mean (sd)	1.63 (0.36)	—	—
UK Donor Liver Index, mean (sd)	1.15 (0.26)	—	—
**Recipient characteristics**		**Mean survival (sd)**	**P-value**
Sex (%):			0.91
Male	73.54	4.55 (0.05)	
Female	26.46	4.56 (0.08)	
Cancer (%):			0.29
yes	29.00	4.62 (0.07)	
no	71.00	4.53 (0.05)	
Decompensated cirrhosis (%):			**<0.001**
yes	37.74	4.73 (0.06)	
no	62.26	4.46 (0.05)	
Non-cirrhotic liver disease (%):			0.63
yes	1.41	4.63 (0.30)	
no	98.59	4.48 (0.04)	
Emergency (%):			**<0.001**
yes	6.72	3.78 (0.17)	
no	93.28	4.58 (0.04)	
MELD exception (%):			0.36
yes	18.00	4.45 (0.09)	
no	82.00	4.57 (0.04)	
Previous transplantation (%):			**<0.001**
yes	8.58	3.76 (0.15)	
no	91.42	4.60 (0.04)	
On dialysis (%):			**<0.001**
yes	5.33	3.43 (0.19)	
no	94.67	4.58 (0.04)	
Medical condition before LT (%):			**<0.001**
Intensive care unit	17.70	3.88 (0.11)	
Hospital (no intensive care unit)	13.88	4.60 (0.10)	
Not hospitalized	68.42	4.71 (0.04)	
Hepatitis B core antibody (%):			0.73
+	20.05	4.53 (0.09)	
−	79.95	4.56 (0.04)	
Hepatitis C virus antibody (%):			**<0.001**
+	23.83	4.23 (0.08)	
−	76.17	4.66 (0.04)	
Diabetes (%):			**0.06**
yes	22.67	4.42 (0.08)	
no	77.33	4.60 (0.04)	
Encephalopathy (%):			**<0.001**
grade 1	68.14	4.62 (0.05)	
grade 2	24.84	4.52 (0.08)	
grade 3	7.02	3.95 (0.17)	
ABO group (%):			0.80
A	45.44	4.56 (0.06)	
B	11.16	4.63 (0.11)	
O	39.03	4.55 (0.06)	
AB	4.37	4.43 (0.19)	
Age, mean (sd)	53.24 (10.39)		
Body mass index (%):			**0.20**
<18.5	3.56	4.35 (0.21)	
18.5–25	44.69	4.50 (0.06)	
≥25	51.75	4.62 (0.05)	
Model for end stage liver disease before LT (%):			**<0.001**
<29	72.73	4.65 (0.04)	
≥29	27.27	4.32 (0.08)	
Waiting time (in days) (%):			**<0.001**
<21	24.54	4.34 (0.08)	
≥21	75.46	4.63 (0.04)	
Follow-up (years), mean (sd)	2.33 (1.63)		

In addition, Table 2 contains formatting errors. The correct Table 2 appears below:

**Table 2:** Retained covariates in the different selection way.

	Covariates	Model 1	Model 2	Model 3	Final model
Donor	**Age**	×	×	×	×
	**Cause of death**	×	×	×	×
	**Intensive care unit stay**	×	×	×	×
	ABO group	×			× (adjustment)
	MDRD creatinine clearance: lowest	×			×
	Partial/split liver	×			×
	Alcohol	×			
	Alkanine phosphatases: latest		×		
	Estimated distance			×	
Recipient	**Re-transplantation**	×	×	×	×
	**On dialysis**	×	×	×	×
	**Medical condition before LT**	×	×	×	×
	**Hepatitis C virus antibody**	×	×	×	×
	**Diabetes**	×	×	×	×
	**Decompensated cirrhosis**	×	×	×	×
	MELD exception	×			×
	ABO group	×			×
Perfomances	C-index	0.626(0.009)	0.616(0.009)	0.616(0.009)	0.625(0.009)
	K statistic	0.617(0.007)	0.611(0.008)	0.610(0.008)	0.616(0.007)
	${R}_{D}^{2}$	0.518(0.020)	0.492(0.021)	0.492(0.021)	0.516(0.021)

Covariates in bold are those which appeared in the three selection models. Model 1: A complete model with selection according to the Akaiké criterion; Model 2: (1) log rank tests with a threshold of 20%, (2) multivariate model included the selected covariates with a selection threshold set at 20%; Model 3: (1) log rank tests with a threshold of 20%, (2) two multivariate models for donor and recipient with the selected covariates with a selection threshold set at 20%, (3) multivariate model with all the covariates selected with a selection threshold set at 20%. The retained full model included all the variables present in at least two of the three models and the set of covariates retained in addition.

Finally, there are errors in Table 5, where the formatting of the “Donor characteristics” header is incorrect. Additionally, the word “Hospital” is incorrectly given as “Hosptial” and the P-values for “ICU stay (in days)” and “Clearance MDRD: lowest (ml/min/1.73 m2)” are omitted. The correct Table 5 appears below as Table 3:

**Table 3:** Differences in donor and recipient characteristics between the derivation and validation datasets (P-values, ${\chi }^{2}$ tests and ANOVA when appropriate).

Recipient characteristics	2009–2013 (n = 3961)	2014 (n = 1048)	P- values
Expert component (no)	3248 (82%)	874 (83.4%)	0.31
ABO group			0.59
A	1800 (45.44%)	465 (44.37%)	
AB	173 (4.37%)	38 (3.63%)	
B	442 (11.16%)	118 (11.26%)	
O	1546 (39.03%)	427 (40.74%)	
Re-transplantation (no)	3621 (91.42%)	961 (91.7%)	0.82
On dialysis (no)	3750 (94.67%)	987 (94.18%)	0.58
Medical condition before LT			0.45
Home	2710 (68.42%)	705 (67.27%)	
Hospital	550 (13.89%)	140 (13.36%)	
ICU	701 (17.7%)	203 (19.37%)	
Hepatitis C virus antibody (−)	3017 (76.17%)	811 (77.39%)	0.43
Diabetes (no)	3063 (77.33%)	793 (75.67%)	0.27
Decompensated cirrhosis (no)	2466 (62.26%)	667 (63.65%)	0.43
**Donor characteristics**	**2009–2013 (n = 3961)**	**2014 (n = 1048)**	**P-values**
ABO group			0.75
A	1766 (44.58%)	460 (43.89%)	
AB	143 (3.61%)	32 (3.05%)	
B	391 (9.87%)	102 (9.73%)	
O	1661 (41.93%)	454 (43.32%)	
Age			**<0.01**
≤69	3029 (76.47%)	710 (67.75%)	
>69	932 (23.53%)	338 (32.25%)	
COD			**<0.01**
Anoxia	476 (12.02%)	150 (14.31%)	
CVA	2388 (60.29%)	651 (62.12%)	
Trauma	976 (24.64%)	231 (22.04%)	
Other	121 (3.05%)	16 (1.53%)	
ICU stay (in days)			0.23
≤4	3156 (79.68%)	853 (81.39%)	
>4	805 (20.32%)	195 (18.61%)	
Clearance MDRD: lowest (ml/min/1.73 m^*2*^)			0.55
<60	1400 (35.34%)	385 (36.74%)	
60–89	1459 (36.83%)	388 (37.02%)	
≥90	1102 (27.82%)	275 (26.24%)	
Liver type (entire)	3759 (94.9%)	1007 (96.09%)	0.13

