# Supplementary material for: A Donor Quality Index for liver transplantation: development, internal and external validation
Source: Sci Rep. 2018 Jun 29;8:9871. doi: 10.1038/s41598-018-27960-7 (PMC6026153; doi:10.1038/s41598-018-27960-7)
Supplement: Supplementary file 1 — Supplementary tables and figures [file 41598_2018_27960_MOESM1_ESM.pdf]

Supplementary Information for A Donor Quality Index for liver transplantation: development, internal and external validation.

Audrey Winter, PhD [1,2,3*], Cyrille Féray, MD PhD [4], Etienne Audureau, MD, PhD [5], Daniel Azoulay, MD, PhD [6], Corinne Antoine MD MSc [7], Jean-Pierre Daurès, MD PhD [1,2], Paul Landais, MD PhD[1]

Supplementary Figures Legends :

- Figure 1S – Calibration plots at month 3, 6 and 12 and their 95 band of the Cox model used to create the Donor Quality Index.
- Figure 2S – Distribution of the Donor Quality Index (DQI) for the derivation and validation datasets.
- Figure 3S – Empirical cumulative distribution functions of the Prognostic Index (PI) by risk group in the derivation and validation datasets.
- Figure 4S – Predicted survival curves in the derivation and validation dataset using the Prognostic Index (PI) and the baseline survival in the derivation dataset (without recipient covariates) with the estimated survival using Kaplan Meier curves in the validation dataset.

Supplementary Tables Legends :

- Table S1 – Reasons for non-collected grafts (2009-2013).
- Table S2 – Reasons for non-transplanted grafts (2009-2013).

Table S1 – Reasons for non-collected grafts (2009-2013).

	**2009**	**2010**	**2011**	**2012**	**2013**
Steatosis	107	107	95	101	89
Chronic alcoholism	73	66	64	72	76
Organ removal denied	54	46	61	69	51
Logistic impediment	39	39	42	43	38
Biology (cytolysis elevated and persistent)	39	29	36	35	34
Hemodynamic instability	19	19	23	20	14
Opposition to organ donation	34	17	16	13	8
Donor age	8	2	2	4	3
Suspicion of tumor	7	5	7	8	5
Traumatism	3	2	4	4	3
Other causes	27	31	53	39	55
Missing causes	8	5	5	0	3
**Total**	**418**	**368**	**408**	**408**	**379**

Table S2 – Reasons for non-transplanted grafts (2009-2013).

	**2009**	**2010**	**2011**	**2012**	**2013**
Macroscopic steatosis (with or without biopsy)	38	41	40	35	28
Poor graft quality	19	23	32	25	17
Tumor (or suspicion)	2	5	9	10	9
Recipient cause (mainly death)	1	1	0	3	1
Other causes*	9	7	4	5	11
**Total**	**69**	**77**	**85**	**78**	**66**

* logistic impediments, damaged graft, technical difficulties of surgery, anatomical difficulties, cardiac arrest during organ removal, ...)

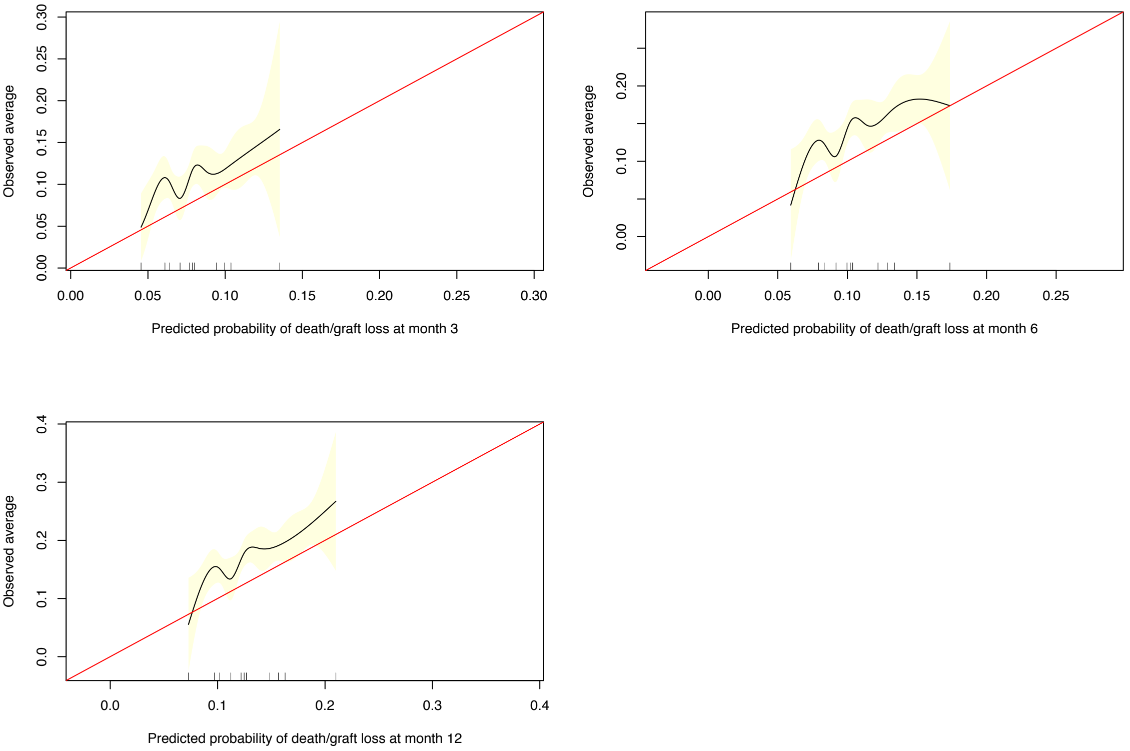

Figure 1S – Calibration plots at month 3, 6 and 12 and their 95 band of the Cox model used to create the Donor Quality Index.

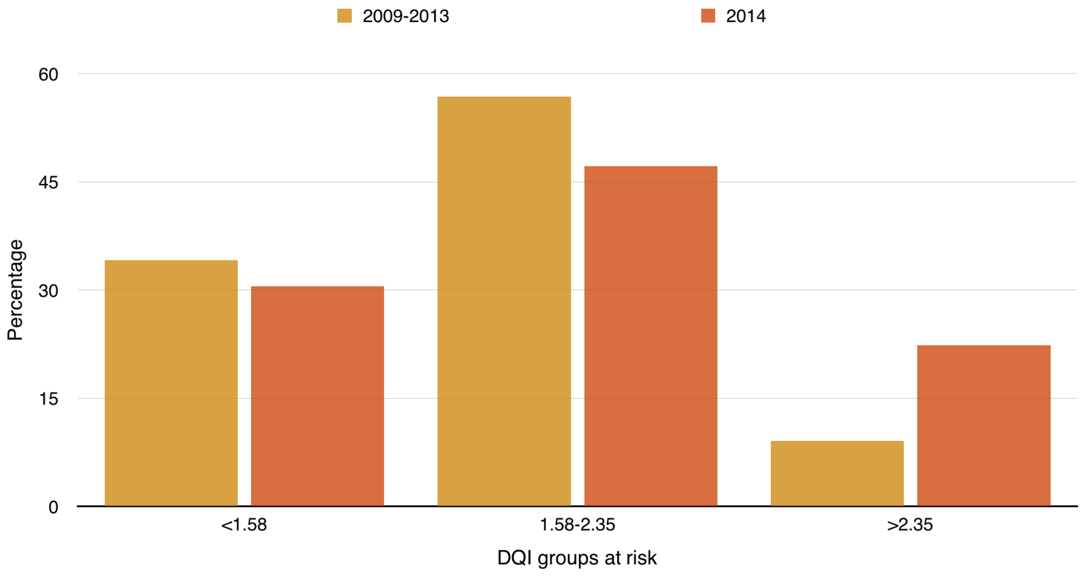

Figure 2S – Distribution of the Donor Quality Index (DQI) for the derivation and validation datasets.

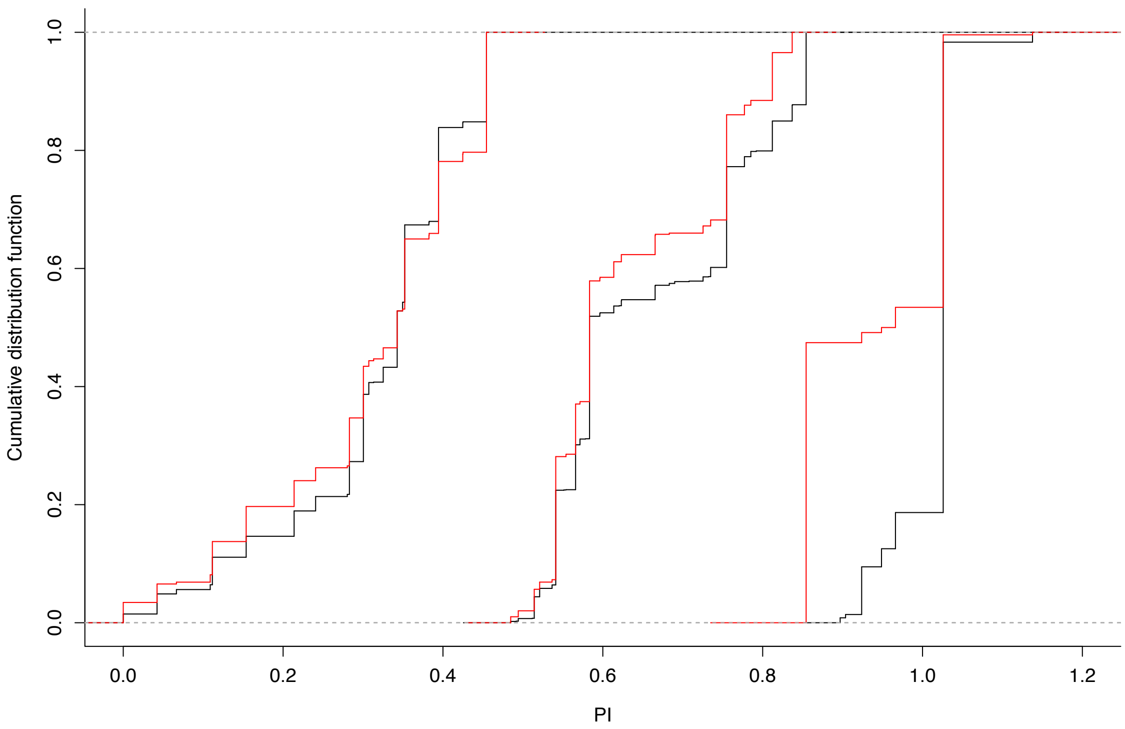

Figure 3S – Empirical cumulative distribution functions of the Prognostic Index (PI) by risk group in the derivation and validation datasets.

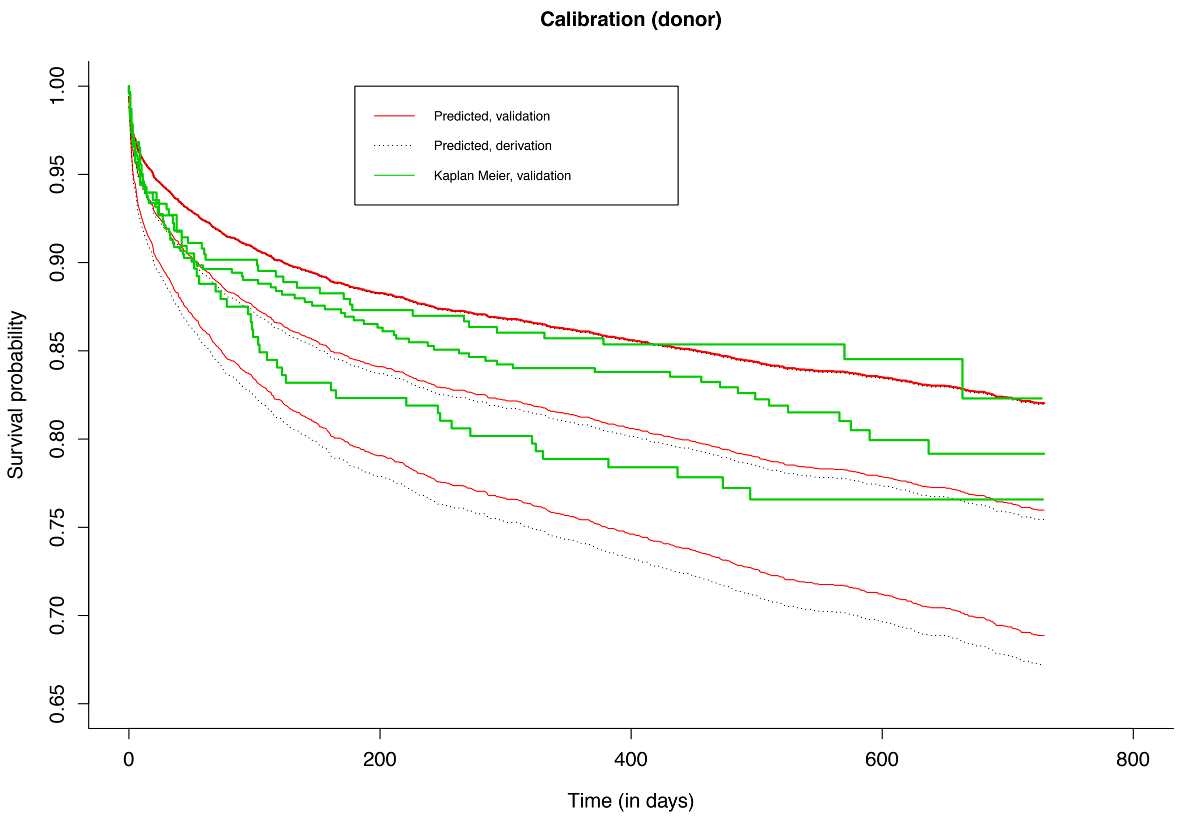

Figure 4S – Predicted survival curves in the derivation and validation dataset using the Prognostic Index (PI) and the baseline survival in the derivation dataset (without recipient covariates) with the estimated survival using Kaplan Meier curves in the validation dataset.
